# Online Mindfulness Training Increases Well-Being, Trait Emotional Intelligence, and Workplace Competency Ratings: A Randomized Waitlist-Controlled Trial

**DOI:** 10.3389/fpsyg.2020.00255

**Published:** 2020-02-21

**Authors:** Ruby Nadler, Julie J. Carswell, John Paul Minda

**Affiliations:** ^1^SIGMA Assessment Systems, Inc., London, ON, Canada; ^2^Department of Psychology, Western University, London, ON, Canada

**Keywords:** mindfulness, mindfulness based intervention, online, resilience, stress, workplace, emotional intelligence, 360 assessment

## Abstract

A randomized waitlist-controlled trial was conducted to assess the effectiveness of an online 8-week mindfulness-based training program in a sample of adults employed fulltime at a Fortune 100 company in the United States. Baseline measures were collected in both intervention and control groups. Following training, the intervention group (*N* = 37) showed statistically significant increases in resilience and positive mood, and significant decreases in stress and negative mood. There were no reported improvements in the wait-list control group (*N* = 65). Trait mindfulness and emotional intelligence (EI) were also assessed. Following the intervention mindfulness intervention participants reported increases in trait mindfulness and increases on all trait EI facets with the exception of empathy. The control group did not report any positive changes in these variables, and reported reductions in resilience and increases in negative mood. Finally, both self and colleague ratings of workplace competencies were collected in the intervention group only and provided preliminary evidence that mindfulness training enhanced performance on key leadership competencies including competencies related to decisiveness and creativity. The present study demonstrates the effectiveness of an online-based mindfulness training program for enhancing well-being, self-perceptions of emotional intelligence, and workplace performance.

## Introduction

Mindfulness-based training programs are gaining traction in the workplace. Organizations including Aetna, Dow Chemical, General Mills, Goldman Sachs, Google, Intel, Nike, SAP, Target, and the United States Marine Corps have implemented mindfulness-based training for the purpose of reducing stress, enhancing employee well-being, and increasing productivity (Jha et al., 2010; Wolever et al., 2012; Aikens et al., 2014; Gelles, 2015). Given the popularity of these programs, there is continued need to empirically validate their efficacy in workplace settings, as noted in a recent review (Lomas et al., 2017).

The growing prevalence of mindfulness programs in organizational settings is due in part to the increasing body of work spanning several diverse research areas of showing benefits of mindfulness-based practices on well-being and performance. Mindfulness-based practices have broadly been found to have several benefits including, but not limited to, reducing stress, anxiety, and depression, and enhancing attentional focus, working memory capacity, cognitive flexibility, positive mood, resilience, immune functioning, interpersonal relationships, and well-being (Astin, 1997; Brown and Ryan, 2003; Davidson et al., 2003; Carson et al., 2004; Tang et al., 2007; Hofmann et al., 2010; Brown et al., 2012; Colzato et al., 2012; Eberth and Sedlmeier, 2012; Ostafin and Kassman, 2012; Mrazek et al., 2013; Roeser et al., 2013; Kersemaekers et al., 2018; Slutsky et al., 2019). Some neuroscientific research has shown that mindfulness meditation may even lead to changes in brain structure and function in regions associated with meta-awareness, body awareness, self-regulation, emotion regulation, attention, and memory (Fox et al., 2014).

Mindfulness is a multifaceted construct, consisting of the observation of moment-to-moment experiences in a non-judgmental, non-reactive, curious manner, along with acting with awareness and intention (Kabat-Zinn, 1990, 2003; Bishop et al., 2004; Shapiro et al., 2006; Brown et al., 2007). This non-judgmental relationship with one’s present moment experience can be cultivated moment-by-moment in one’s daily life or during formal meditation practice (Brown and Ryan, 2003, 2004; Glomb et al., 2011). The practice of mindfulness meditation is believed to exert its beneficial effects by increasing awareness of, and attentional control over, one’s present-moment experience (Bishop et al., 2004; Jha et al., 2007; Lutz et al., 2008), strengthening self-regulatory capacities (Vago and Silbersweig, 2012), including emotion regulation capacities (Baer, 2003; Brown and Ryan, 2003; Goldin and Gross, 2010; Jimenez et al., 2010; Vago and Silbersweig, 2012). One’s experience of the present moment is “decentered,” shifting from a closely fused personal identification with thoughts and feelings to a broader awareness, with space between thoughts, feelings, and reactions for more flexible thought and behavioral patterns to emerge (Bishop et al., 2004; Shapiro et al., 2006).

Mindfulness-based interventions have been conducted in clinical and non-clinical settings (see Chiesa and Serretti, 2009; Khoury et al., 2015 for meta-analyses), and are increasingly being conducted in organizational settings. Organizational psychologists have argued that mindfulness should have beneficial effects on employees by increasing awareness and self-regulation, positively impacting workplace performance, relationships, and well-being (Glomb et al., 2011; Dane and Brummel, 2013; Hyland et al., 2015; Good et al., 2016). However, many researchers have called for more research on mindfulness in applied settings (Glomb et al., 2011; Dane and Brummel, 2013; Good et al., 2016; Creswell, 2017; Lomas et al., 2017). Lomas et al. (2017) noted that few high-quality mindfulness interventions have been conducted in organizational settings, as opposed to healthcare and educational settings. More broadly in a recent review Eby et al. (2017) noted that less than one of third of mindfulness intervention studies used a randomized waitlist control group, with most using a pre-test/post-test design with no control group, making it hard to draw conclusions about the potential benefits of mindfulness interventions. The aim of the present study was to assess the potential benefits of an online mindfulness-based intervention with a group of highly educated and skilled knowledge workers using a randomized, waitlist control design, using both self-report and other ratings of workplace effectiveness, with the intention of providing a fuller picture of the potential benefits of an online mindfulness-based intervention in the workplace.

### Prior Mindfulness Intervention Literature and Current Study Hypotheses

#### Mindfulness Interventions and Trait Mindfulness

Prior mindfulness-based interventions have shown that self-reported trait mindfulness increases following intervention in both clinical and non-clinical samples. However different assessments have been used in prior research. Some research has employed a measure of trait mindfulness, such as the Mindful Attention Awareness Scale (MAAS; Brown and Ryan, 2003), that treats mindfulness as a unidimensional construct, and shown increases in this scale following a mindfulness-based intervention (Ortner et al., 2007; Klatt et al., 2009; Hülsheger et al., 2013). Other research has employed a multifaceted measure of mindfulness, such as the Five Facet Mindfulness Questionnaire (FFMQ; Baer et al., 2006) or the Five Facet Mindfulness Questionnaire Short Form (FFMQ-SF; Bohlmeijer et al., 2011). Aikens et al. (2014) used the FFMQ as a trait mindfulness outcome measure and reported increases on all of the facets. Querstret et al. (2018) used 4 of the 5 facets of the FFMQ-SF (the observing facet was excluded due to prior research by Baer et al. (2006, 2008) showing that observing didn’t load onto a single mindfulness construct and did not show changes in meditation-naïve participants), and reported that the non-reactivity facet did not change following the interventions. Finally, several other interventions did not report the use of any mindfulness assessment as an outcome measure (Davidson et al., 2003; Bazarko et al., 2013; Chin et al., 2019b; Slutsky et al., 2019), preventing conclusions about the effects of those intervention on trait mindfulness to be drawn. In the present research we elected to employ the FFMQ-SF, and hypothesized that the mindfulness facets would increase following the mindfulness intervention relative to a randomized waitlist control group (H1).

#### Mindfulness Interventions and Well-Being

The finding that mindfulness interventions reduce psychological distress and enhance well-being has been well established. For instance, Davidson et al. (2003) conducted an on-site mindfulness-based intervention using the 8-week Mindfulness-Based Stress Reduction (MBSR; Kabat-Zinn, 1982, 1990) program in a high-stress workplace and reported reductions in anxiety and negative mood, and improvements in immune function. Other interventions and samples have reported reductions in self-reported perceived stress (Klatt et al., 2009; Wolever et al., 2012; Bazarko et al., 2013; Malarkey et al., 2013; Aikens et al., 2014; Chin et al., 2019a, b). Some research studies assessing perceived stress screened out participants with lower levels of self-reported stress prior to the intervention (Wolever et al., 2012; Chin et al., 2019a), however other interventions that did not prescreen participants based on stress levels also reported significant reductions in perceived stress (Bazarko et al., 2013; Malarkey et al., 2013; Aikens et al., 2014), leading us to predict that the present mindfulness intervention would also lead to significant reductions in self-reported perceived stress relative to a randomized waitlist control group (H2) (without prescreening participants based on their baseline stress levels).

Prior interventions have looked at a range of other well-being outcome measures including burnout, resilience, vitality, and vigor, often using participants working in healthcare, such as physicians and nurses, and have reported that mindfulness interventions reduce burnout, and increase resilience, vigor, and vitality (Galantino et al., 2005; Mackenzie et al., 2006; Krasner et al., 2009; Bazarko et al., 2013; see Virgili, 2015 for a meta-analysis). We were interested in whether employees in a non-healthcare setting, who were also not employed in the service industry (which can require large amounts of emotion regulation and surface acting, e.g., Michel et al., 2014; Hülsheger et al., 2013) would show improvements in self-reported resilience following the mindfulness intervention. We predicted that mindfulness intervention participants would show enhanced resilience following the intervention (H3).

Previous research has reported increases in positive mood and decreases in negative mood following mindfulness interventions (Ortner et al., 2007; Chin et al., 2019b), although some studies failed to report increases in positive mood, but reported decreases in negative mood (Davidson et al., 2003). We predicted that people would report higher levels of positive mood, and lower levels of negative mood following the mindfulness intervention (H4). Based on prior research, we expected to find larger reductions in negative mood relative to increases in positive mood (H4a) (Brown and Ryan, 2003; Davidson et al., 2003; Chambers et al., 2008).

#### Mindfulness Interventions and Emotional Intelligence

Emotional intelligence (EI), like mindfulness, is a multifaceted construct, and can be broadly defined as “the ability to monitor one’s own and others’ feelings and emotions, to discriminate among them and to use this information to guide one’s thinking and actions” (Salovey and Mayer, 1990, p. 189). Some researchers operationalize EI as an *ability* relying on cognitive processes, whereas others operationalize it as a personality-based disposition or *trait* that is relatively stable, and some researchers argue for a mixed approach where traits and abilities are both accounted for, based on the reasoning that there must be some pre-existing inclination in an individual to pay attention to emotionally laden information before ability-based action can be taken (Tett et al., 2005, 2006). The various ways EI has been operationalized necessitate the development of different EI assessments, and a plethora of diverse assessment options exist. Ability-based measures are often based on scenarios requiring the use of emotional information and have clear right or wrong answers. Trait-based measures use self-report assessments. Research has shown that both types of EI are related to mindfulness. Snowden et al. (2015) reported that nurses with prior mindfulness training demonstrated greater ability EI but not greater trait EI. Positive relationships between trait mindfulness and self-reported trait EI have been reported using a variety of measures (Brown and Ryan, 2003; Baer et al., 2004, 2006; Cohen and Miller, 2009; Bao et al., 2015), and some of the benefits associated with mindfulness, such as the positive relationship between self-reported mindfulness and life satisfaction, have been shown to be mediated by trait EI (Schutte and Malouff, 2011; Wang and Kong, 2014). EI has also been targeted as a critical skill in the workplace, with research showing positive relationships between trait EI and work engagement (Schutte and Loi, 2014), and performance at work (Joseph and Newman, 2010; O’Boyle et al., 2011).

Despite these associations and the importance of EI in the workplace, few studies have looked directly at the influence of mindfulness practices on EI in organizational settings. In the present research, we used a multifaceted measure of self-perceived emotional intelligence specific to workplace situations, the Multidimensional Emotional Intelligence Assessment - Workplace (MEIA-W; Tett et al., 2006) to find out whether people’s self-perceived trait emotional intelligence would change following a mindfulness intervention. We predicted that trait EI scores would increase in mindfulness participants relative to control participants (H5). Specifically, we expected that self-perceptions of recognition and regulation of emotions in the self would increase following the intervention due to prior research linking trait mindfulness with attention to emotions and clarity of emotions (Brown and Ryan, 2003). Further, if mindfulness enhances the ability to pay attention as evidenced by Jha et al. (2007) and based on accounts of how mindfulness interventions enhance attention and awareness (Shapiro et al., 2006), we expected that the intervention could also increase self-perceived recognition of emotion in others and potentially self-perceived regulation of emotion in others. Prior research on the impact of mindfulness interventions on self-reported empathy has been mixed (Shapiro et al., 1998; Beddoe and Murphy, 2004; Galantino et al., 2005), so our inclusion and examination of this component was more exploratory, as was the inclusion of self-perceived non-verbal emotional expression. It is possible that some interventions highlight the importance of empathy more than others, for instance, those conducted in healthcare with “helping” occupations such as nurses may be more successful in enhancing empathy than those conducted in office-based organizational settings.

#### Mindfulness Interventions and Job Performance

Some of the mindfulness interventions conducted in organizational settings have assessed work/life balance and work performance using both self and other ratings. Most prior research has shown improvements on perceived work/life balance (Mackenzie et al., 2006; Allen and Kiburz, 2012; Michel et al., 2014). However, when looking at productivity, results have been limited and mixed. Wolever et al. (2012) did not report any improvements in productivity, whereas Slutsky et al. (2019) reported that employees felt more focused and productive following an on-site mindfulness intervention but did not collect any data that was not based in self-assessment. Shonin et al. (2014) conducted an intervention in office-based employees that resulted in lower levels of stress, psychological distress, and higher levels of job satisfaction and supervisor-rated workplace performance. Finally, a recent study by Bartlett et al. (2017) looked at the influence of a mindfulness intervention on informant-ratings and reported some indication via qualitative reports that the program had benefits observable to outsiders, but quantitative results did not mirror this finding.

In the current research we elected to assess both self-ratings of several workplace competencies, including work/life balance, as well as peer-assessment of the same workplace competencies. Due to limited prior research, the following hypotheses are tentative. However, if the intervention improves participants’ mood and resilience, and reduces stress and negative mood, as predicted (H2–H4), there is also support for the notion that self-reported workplace performance will generally improve (Estrada et al., 1997; Lyubomirsky et al., 2005; Fredrickson et al., 2008). In particular we expected ratings related to higher level thinking processes, such as decisiveness and creativity, to improve due to the known link between mindfulness and cognitive flexibility (Colzato et al., 2012; Ostafin and Kassman, 2012), H6, which should be related to both decisiveness and creativity. We also hypothesized that there should be improvements in competencies related to working with others (Carson et al., 2004), H7, such as interpersonal relationships. Although we had the aforementioned targeted hypotheses, we employed a wider range of 27 workplace competencies relevant to the job requirements in our sample population as an exploratory first step due to the lack of prior research. Finally based on prior research by Shonin et al. (2014) showing improved workplace performance ratings, we hypothesized that colleague raters would provide higher ratings for intervention participants following the mindfulness intervention (H8).

#### Mindfulness Intervention Format (In-Person Versus Online)

Prior mindfulness interventions have been conducted using in-person and online delivery formats. The benefits of in-person instruction for mindfulness practices are plentiful, but in-person instruction can be costly and time intensive. With employees increasingly located in different parts of the world working under different priorities and schedules, remote delivery of mindfulness instruction is an attractive, cost-effective option. Prior research has shown that online mindfulness instruction is as effective as in-person instruction (Wolever et al., 2012; Bazarko et al., 2013; Aikens et al., 2014; Querstret et al., 2018). For the present research we elected to employ an online mindfulness training program for the mindfulness intervention that could be accessed from any internet-connected device (smartphone, computer, or tablet), at any time to accommodate different employee participant schedules and time constraints. Although the program did not allow for real-time interaction between the mindfulness facilitator and participants because the instructional materials were pre-recorded, participants were able to contact the facilitator through the online program portal or via email.

#### Length of Intervention and Amount of Mindfulness Practice

What constitutes a “low” dose of mindfulness training has varied across different studies and was another consideration for the present research. For example, Slutsky et al. (2019) employed a half-day mindfulness workshop for their “low dose” mindfulness control condition, whereas Klatt et al. (2009) conducted a “low dose” intervention consisting of 6 weeks of instruction and 20 min of daily practice. However, in comparison to the original MBSR course, which includes 2.5–3 h of weekly instruction and 1 h of daily practice over 8 weeks as well as a day-long instructor-led mindfulness retreat, most interventions that do not employ the full MBSR curriculum have contained less instruction and lower prescribed amounts of mindfulness practice in comparison to a MBSR intervention. A review of mindfulness interventions revealed an array of lengths and daily prescribed practices: Hülsheger et al. (2013) employed a 2-week self-taught mindfulness training intervention that required participants to complete a 3-min breathing meditation twice daily in addition to longer practices; Querstret et al. (2018) employed a 4-week online mindfulness intervention that included 20–30 min of daily practice; Slutsky et al. (2019) conducted a 6-week in-person intervention that required 25 min of daily practice; Aikens et al. (2014) employed a 7-week online intervention prescribing 20 – 45 min of daily practice; Malarkey et al. (2013) employed an 8-week intervention that required 1 h of weekly instruction in addition to 20 min of daily practice; Wolever et al.’s (2012) mindfulness intervention was 12-weeks long and consisted of 14 h of instruction and 5–15 min of daily practice presented in both in-person and online sessions. While the length of the interventions and amount of daily practice prescribed varied across studies, prior research has shown that the length of the intervention, length of daily practice, and delivery format can vary and still have a positive impact on employee outcome measures. We elected to employ an 8-week mindfulness-based intervention format that prescribed a minimum of 3 min a day of mindfulness practice, but included longer practice sessions depending on the week, and guided practice selected by the participant. This is a lower amount of daily practice than the interventions reviewed above, but we wanted to emphasize consistent daily practice over length of practice and felt committing to a short daily practice would be less intimidating and more feasible even for the busy professionals in our participant sample compared to a 20 or 30-min daily requirement.

## Materials and Methods

### Participants

**FIGURE 1:**
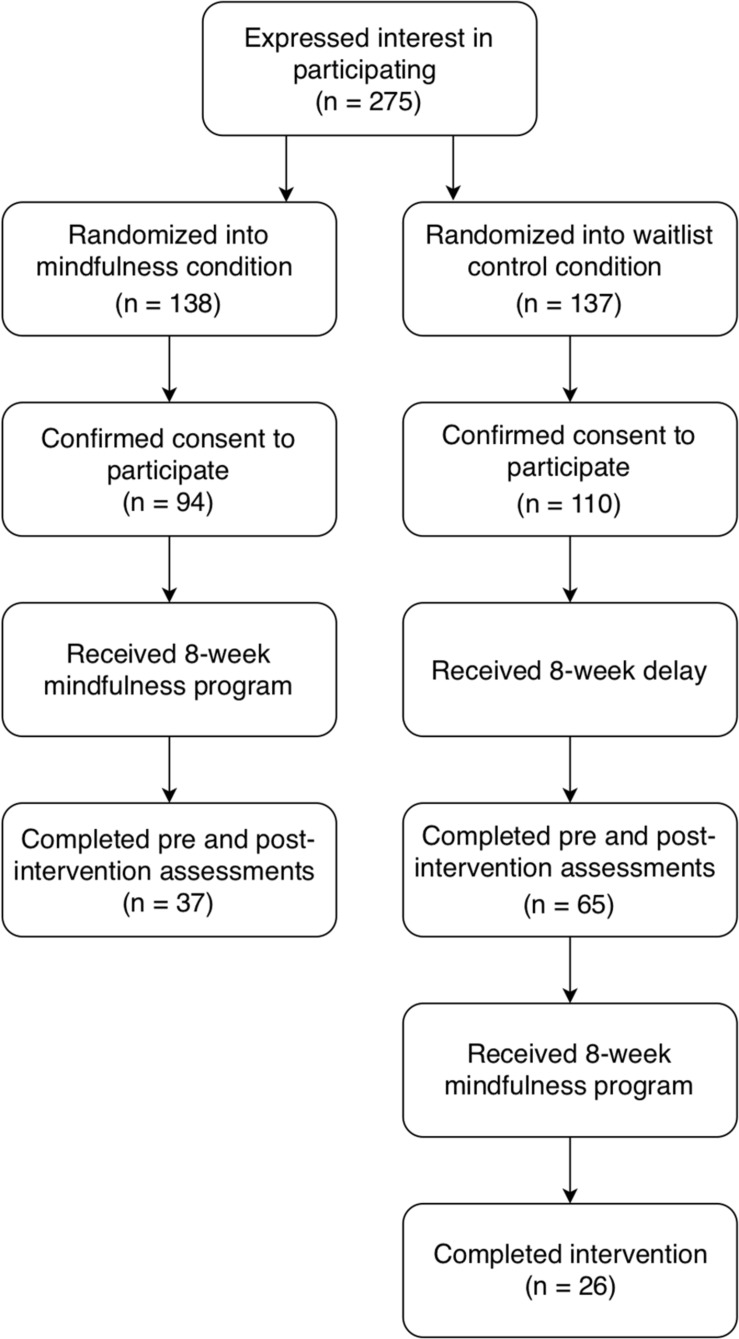
Participant flowchart.

Two hundred and eighty nine employees employed by a US-based Fortune 100 company expressed interest in the study following a presentation made available to employees throughout the company. Participants were eligible for participation in the study if they were fluent in English, able to access the online program via an internet-connected device (e.g., smartphone, computer, or tablet), and if they were a US-based, fulltime employee of the company hosting the study. Figure 1, the participant flowchart, shows the randomized waitlist control procedure and attrition rates.

### Randomization

The eligible participant sample (*N* = 275) was randomly assigned to the mindfulness intervention condition (*n* = 138) or the control condition (*n* = 137). Success of the randomization procedure was evaluated using a Pearson Chi-square test of significance on the randomized sample who completed baseline assessments (*n* = 204). No significant differences between the intervention and waitlist control groups were found for age, sex, and prior meditation experience (all *p*’s > 0.05), confirming that the randomization was successful.

### Participant Demographics

Of the 138 individuals assigned to the mindfulness intervention condition, 90 participants completed the baseline assessment and 37 completed the post-assessment, resulting in an attrition rate of 59%. Of the participants assigned to the control group, 110 consented to participate, 103 completed the baseline assessment, and 65 waitlist participants completed the post-assessment, an attrition rate of 37%. There were no significant differences in baseline demographics (age, sex, hours of work, prior meditation experience) or between pre-intervention outcome measures (perceived stress, mood, resilience, trait mindfulness, perceived emotional intelligence tendencies) between participants who completed the study and those who did not, (all *p*’s > 0.05). Of the participants who completed baseline and post-intervention assessments (*N* = 102), 73.5% of the participants were female (26.5% male), ranged in age from 18 to 60+ years (63.8% were between 40 and 59 years of age), and most (89.2%) held a university degree or higher education. Ethnicity data was not collected. Forty-six percent of the sample indicated they had some prior meditation experience or exposure. Of participants with prior experience, 23.5% had a year or less of experience and 15.7% had 1–3 years of experience. Of those who indicated that they had prior mindfulness experience, a minority (17.6%) reported practicing meditation three times per week or more. There was a significant difference between the mindfulness and control condition participants when the gender of participants was examined, with significantly fewer males in the mindfulness group than in the control group, *x*^2^(1) = 5.01, *p* < 0.05, indicating that more males than females didn’t complete the second assessment. Twelve of the mindfulness condition participants (9 males) who did not complete the post-assessment recorded between 2 and 10 h of meditation practice over the 8-week program, making it unlikely that they had dropped out of the program. No other significant differences existed between intervention and control participant groups at baseline on the demographic variables, all *p*’s > 0.05. The full demographics of study participants is shown in Table 1.

**TABLE 1:** Participant demographic information.

	Mindfulness (*n* = 37)		Control (*n* = 65)	
	*Count*	*%*	*Count*	*%*
**Gender**				
Male	5	13.5	22	33.8
Female	32	86.5	43	66.2
**Age**				
18–29	1	2.7	2	3.1
30–39	4	10.8	13	20.0
40–49	13	35.1	19	29.2
50–59	13	35.1	20	30.8
60+	6	16.2	8	12.3
Prefer not to say	0	–	3	4.6
**Education**				
High school/GED	0	0	1	1.5
Some college	0	0	3	4.6
College	2	5.4	4	6.2
University	20	54.1	31	47.7
Masters	8	21.6	15	23.1
Doctorate	4	10.8	8	12.3
Professional degree	3	8.1	2	3.1
Prefer not to say	0	0	1	1.5
**Hours worked**	***M***	***SD***	***M***	***SD***
Average hours worked per week	44.73	5.55	44.02	8.12
**Prior Meditation Experience**				
Yes	19	51.4	28	43.1
No	18	48.6	37	56.9
**Length of practice**				
Less than one month	6	16.2	2	3.1
1–3 months	1	2.7	5	7.7
3–6 months	2	5.4	3	4.6
6–12 months	1	2.7	4	6.2
1–3 years	7	18.9	9	13.8
3+ years	3	8.1	6	9.2
N/A	17	45.9	36	55.3
**Frequency of practice**				
1–2x daily	1	2.7	2	3.1
1–2x weekly	6	16.2	4	6.2
3 or more times/week	4	10.8	1	1.5
A few times/month	1	2.7	10	15.4
Other	6	16.2	4	6.2
N/A	19	51.4	43	66.2

### Intervention: Online Workplace-Based Mindfulness Training

The current intervention consisted of an online 8-week mindfulness-based program developed by SIGMA Assessment Systems Inc, based on Dr. Jon Kabat-Zinn’s mindfulness-based stress reduction (MBSR) program (Kabat-Zinn, 1982, 1990) and the mindfulness-based cognitive therapy (MBCT) program (Segal et al., 2002). The program presented mindfulness information and techniques in an online format. An outline of the content can be seen in Table 2. Content consisted of short videos (6–12 min long), brief guided meditation practices (3–20 min long with an average length of 10 min), and suggestions for how to integrate mindfulness into daily activities at work. Participants received a weekly email introducing that week’s theme and content, and were directed from that email to login to the program platform. Participants were asked to watch the weekly video and practice the guided meditations 6 out of 7 days a week (for a total of 144 – 480 min depending on the length of the meditation practice). A meditation tracker allowed participants to log the date, length of practice, and time of day (morning, afternoon, evening, or overnight) they completed a meditation and participants were encouraged to use the tracker. Participants could access the program on any internet-connected compatible device (i.e., smartphone, computer, or tablet) and could access the program 24 h a day while at work or at home^1^.

**TABLE 2:** Eight-week mindfulness program outline.

Week	Topic	Guided Meditation Practice	Attitude/Quality
1	Foundations of Mindfulness	3-min breath-based	Non-judgment
2	The Mind-Body Connection	10-min body scan	Curiosity
3	Motivation and Communication	10-min breath-based	Non-striving
4	Emotional Intelligence	10-min breath-based	Gratitude
		Growing good feelings	
5	Slow Brain, Fast Brain	10-min open-monitoring	Beginner’s Mind
6	Creativity and Innovation	10-min open-monitoring	Awe
7	Judgment and Decision-Making	10-min breath-based	Perspective
		Perspective taking	
8	Moving Forward with Mindfulness	20-min breath-based 10-min loving-kindness practice	You at your best reflection

### Ethics, Data Storage, Anonymity

Participants provided informed written consent. Study procedures were approved by the university’s institutional ethics review board and followed APA ethics guidelines for research with human participants. Participants were provided with access to the online mindfulness program but were not otherwise compensated for their participation.

Pre- and post-assessment data was collected using Qualtrics. The online program portal stored participant login information as well as meditation tracker data specific to each participant. Both services encrypted data in transit (HTTPS) and the program portal stored data in encrypted form. Data downloaded from both services were stored on a password protected server that was only accessible to those associated with the study.

Due to the nature of the multisource rater analyses, fully anonymizing the data at the time of data analysis was not possible because of the need to match multisource colleague ratings with specific intervention participants. Identifying information was removed after the cases were matched so that datasets contain only anonymized participant IDs at the present time.

### Outcome Measures

#### Five Factor Mindfulness Questionnaire – Short Form (Baer et al., 2012)

The 24-item Five Factor Mindfulness Questionnaire – Short Form (FFMQ-SF) uses a 5-point Likert scale ranging from 1 “Never or very rarely true” to 5 “Very often or always true.” It assessed five factors of mindful awareness: *Observing* internal and external sensations (4 items, present sample α = 0.76 – 0.82, example item “I pay attention to physical experiences, such as the wind in my hair or sun on my face”), *Describing* internal experiences (5 items, present sample α = 0.84 – 0.87, example item “I’m good at finding the words to describe my feelings”), *Acting with awareness*, in contrast to unintentional behavior, e.g., autopilot (5 items, present sample α = 0.77 – 0.85, example item “I rush through activities without being really attentive to them”), *Non-judging* of inner experience (5 items, present sample α = 0.80 – 0.88, example item “I tell myself that I shouldn’t be feeling the way I’m feeling”), and *Non-reactivity* to inner experience (5 items, present sample α = 0.77 – 85, example item “I watch my feelings without getting carried away by them”). The summed range of each subscale is between 5 and 25 (5 and 20 for Observing). This measure is sensitive to changes in mindfulness as a result of intervention (Bohlmeijer et al., 2011).

#### Perceived Stress Scale (PSS; Cohen et al., 1983)

The 14-item PSS (present sample α = 0.88 – 0.90) consists of items that assess how often one has experienced stress in the past month, example item “In the past month, how often have you found that you could not cope with all the things you had to do?”. It is scored on a 5-point Likert scale ranging from 0 “Never” to 4 “Very Often” with a possible range in scores from 0 – 56. Scale summed scores are reported in the study results.

#### Brief Resilience Scale (BRS; Smith et al., 2008)

The BRS consists of 6 items (present sample α = 0.86 – 0.89) that assess one’s tendency to move past stressful, difficult experiences, example item “It does not take me long to recover from a stressful event.”. It is scored on a 5-point Likert scale ranging from 1 “Strongly disagree” to 5 “Strongly agree,” and has a possible range from 6 to 30. In the results the summed score is divided by the number of questions answered resulting in average scores.

#### Positive Affect Negative Affect Schedule (PANAS; Watson et al., 1988)

The PANAS assesses positive and negative affective dimensions on distinct scales using 10 *Positive* single word descriptor items (i.e., “excited,” “motivated”; present sample α = 0.89 – 0.92) and 10 *Negative* single word descriptor items (i.e., “jittery,” “agitated”; present sample α = 0.87 – 0.90). It is scored using a 5-point Likert scale from 1 “Very slightly or not at all” to 5 “Extremely,” resulting in a possible range of scores from 10 to 50 for each of the positive and negative scales. Summed total scores are reported in the study results. Participants were asked to think of how they felt in the past month when responding.

#### Multidimensional Emotional Intelligence Assessment – Workplace (MEIA-W; Tett et al., 2006)

The MEIA-W assesses all components of the Salovey and Mayer (1990, p. 190) “Four-Branch Model” of emotional intelligence (EI), consisting of *Recognition of Emotion in Self* and *Recognition of Emotion in Others*, *Regulation of Emotion in Self*, and *Regulation of Emotion in Others*. Additionally, the MEIA-W assesses *Empathy* and *Non-verbal Emotional Expression*, as well as 4 “proximal” outcomes of EI (*Intuition vs. Reason*, *Creative Thinking*, *Mood Redirected Attention*, and *Motivating Emotions*). The MEIA-W groups Recognition of Emotion in the Self (“When I get upset at work, I always know the exact cause of it,” present sample α = 0.78 – 0.87); Regulation of Emotion in the Self (“I can keep myself calm even in highly stressful work situations,” present sample α = 0.88 – 0.89); Recognition of Emotion in Others, (“I can tell what my coworkers are feeling even when talking to them over the phone,” present sample α = 0.76 – 0.86); Regulation of Emotion in Others, (“Usually, I know what it takes to turn a coworker’s boredom into excitement,” present sample α = 0.69 – 0.83); as well as Empathy (“If I saw someone at work being harassed, I would get upset,” present sample α = 0.70 – 0.78); and Non-verbal Emotional Expression, („My coworkers would say that, emotionally, I am very easy to read,” present sample α = 0.59 – 0.77), into a “core” group of EI facets, and all 72 items from these 6 EI facets were administered and included in the results. To reduce the time required to complete the assessment, the 48 proximal outcome items were not administered. The MEIA-W uses a 5-point Likert Scale ranging from 1 “Strongly disagree” to 5 “Strongly Agree,” and the present results report the average scores for each facet are presented and used in analyses. The MEIA-W is not an ability-based measure with “correct” and “incorrect” responses, it is intended to assess people’s trait-based EI, that is, their tendency to draw upon emotional intelligence at work. The MEIA-W was designed to reduce social desirability biases as much as possible in a self-report measure by avoiding the use of statements that are overly desirable or undesirable.

#### Workplace Competency Assessment (Jackson, 2003)

A commercial multisource performance rating instrument was used to collect information on 27 job competencies in the intervention group only. The competences are based on Yukl’s (2006) taxonomy and are listed with definitions in Table 3. Competencies were chosen based on the knowledge workers’ specific industry and included competencies including “*Creativity* – Demonstrating the ability to initiate original and innovative ideas, products, or approaches,” and “*Decisiveness* – The ability to make clear-cut and timely decisions with the appropriate amount of information.” Each intervention participant rated themselves on all 27 competencies. Intervention participants also invited multiple colleagues to provide performance ratings. A total of 211 colleagues provided ratings on intervention participants. Instructions were to “Read the description below and rate how effective (you/your colleague) are at performing the behavior” on a 7-point Likert scale ranging from 1 “Low” to 7 “High.” Colleague raters were provided with instructions to encourage accurate ratings and to avoid overuse of the top part of the scale. To further encourage accurate ratings, colleagues were reminded that their ratings were private, would not be shared with the participant they were rating, and that any data reported would include only group averages. The average one-way random intra-class correlation coefficient (ICC) for colleague ratings on the competencies based on ICC (1, 5) is 0.60, indicating adequate inter-rater reliability for interpretation.

**TABLE 3:** Multisource rater feedback competency labels and definitions.

Competency	Definition Provided to Participants and Colleague Raters
Decisiveness	The ability to make clear-cut and timely decisions with the appropriate amount of information.
Creativity	Demonstrating the ability to initiate original and innovative ideas, products, and approaches.
Thoroughness	The ability to attend to detail and develop a comprehensive approach to problems.
Objectivity	The ability to maintain a realistic perspective and keep personal biases to a minimum.
Prioritizing	The ability to quickly identify critical tasks and manage time accordingly to complete these tasks without getting distracted by less important matters.
Mental Agility	Generating multiple solutions to problems quickly and demonstrating the ability to comfortably and easily change topics during conversation and continue to offer penetrating insights.
Intellectual Horsepower	Quickly grasping complex concepts and relationships.
Emotional Depth	Applying a depth of understanding and emotional maturity that allows the appropriate amount of emotion to guide decisions and actions.
Making Tough Calls	Making difficult decisions in a timely manner.
Open-Mindedness	A willingness to consider new ideas and approaches, as well as input from others.
Interpersonal Relations	Relating to others in an outgoing, friendly, warm, and personable manner in order to establish and maintain effective interpersonal relationships.
Social Astuteness	The ability to accurately read and respond diplomatically to organizational trends and norms, as well as effectively deal with organizational politics.
Conflict Management	The ability to mediate and resolve conflicts and disagreements in a manner best for all parties involved.
Communication	Keeping direct reports and leaders informed about decisions, events, and developments that affect them.
Persuasiveness	The ability to sell others on ideas, approaches, products, and services.
Negotiation	The ability to negotiate outcomes that further the interests of the organization, and when possible, also further the interests of opposing groups.
Listening	Taking the time to listen to others’ questions, concerns, and viewpoints, and identifying the relevant information, and conveying it to the other person.
Achievement and Motivation	Demonstrating the motivation to work hard, be successful, achieve difficult goals, and complete challenging tasks.
Independence	The ability to be self-starting and work independently of others when necessary.
Emotional Control	Maintaining personal composure during times of stress or pressure, when things are uncertain, or when faced with conflict or disagreement.
Dependability	The ability to be counted on to meet commitments and deadlines.
Integrity	Demonstrating a high quality of character including being honest, ethical, trustworthy, and sincere, and effectively representing and respecting company values.
Desire to Learn	Embracing new challenges and the opportunity to learn, as well as demonstrating the motivation to grow and develop by responding positively to constructive feedback.
Assuming Responsibility	The willingness to step forward and take charge of a difficult situation, without being asked to do so.
Vision	Seeing the “big picture” in the organization, industry, and economy, including having a clear sense of the company’s ideal future state and communicating this to others in a compelling way.
Productivity	Accomplishing an above average quantity and quality of work.
Work/Life Balance	Maintaining a healthy and productive balance between work responsibilities and life outside of work.

### Statistical Analysis

Analyses presented here include only the data from those participants that completed the full study (e.g., completed all surveys pre- and post-intervention). Data was cleaned, matched, and sorted in Excel and then exported to SPSS (version 26) where data analyses were conducted.

### Power Analysis

A statistical power analysis was conducted using GPower (Erdfelder et al., 1996), to confirm that we had a sufficiently large sample size to detect significant changes in our outcome measures. Twenty five participants total were needed in each group (total sample size = 50) for adequate power (i.e., 0.90 – 0.99) to detect significant differences in our analyses with medium to large effect sizes (0.5 – 1.2). Effect sizes were based on pilot data collected with a separate sample of employees in the same company using the same outcome measures, and we also ran *post hoc* power analyses based on effect sizes from the present study. We did not have sufficient power to detect changes in workplace competency ratings in the sample of colleague raters, but had sufficient power for self-ratings on the workplace competencies using a one-tailed test of significance.

## Results

### Engagement With Program

Thirty intervention participants out of 37 recorded their meditation practices, totaling 8, 748 min of meditation practice (*M* = 4.86 h per participant) over the course of the 8-week program using the online program’s meditation tracker. Participants reported practicing in the morning (53% of the time) and evening (28% of the time) most of the time, followed by the afternoon (15% of the time) and overnight (4%). Participants reported practicing for an average of 9.5 min at a time. The average amount of practice reported by participants falls within the lower recommended range of practice, which was 3–10 min a day 6 out of 7 days a week (for a total of 2.4–8 h over the course of the program).

#### Pre-Post Intervention Changes

**FIGURE 2:**
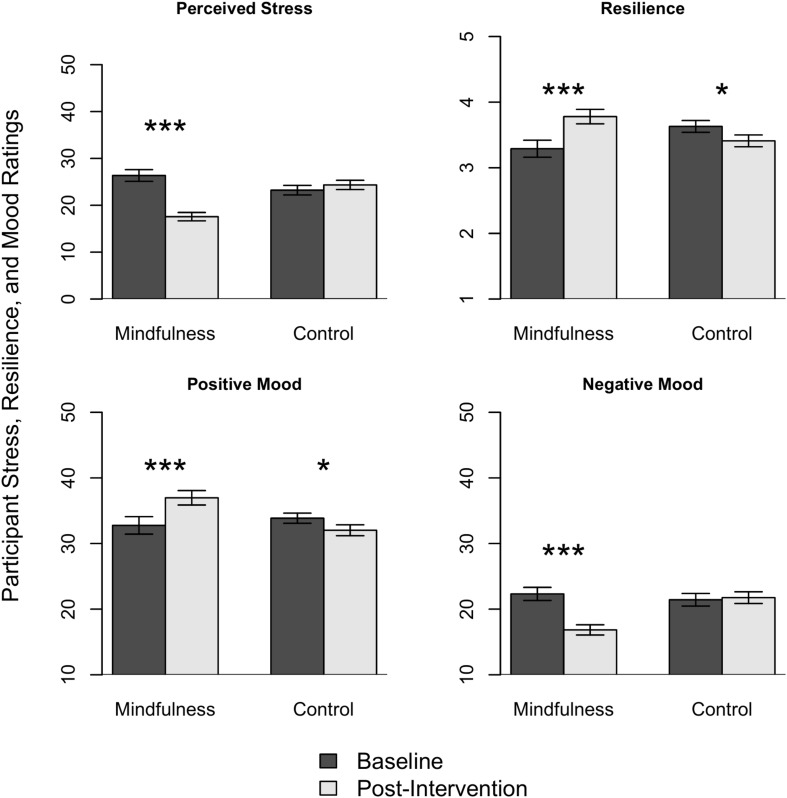
Well-being ratings pre and post-intervention for mindfulness and control groups. *p < 0.05; ***p < 0.001.

**FIGURE 3:**
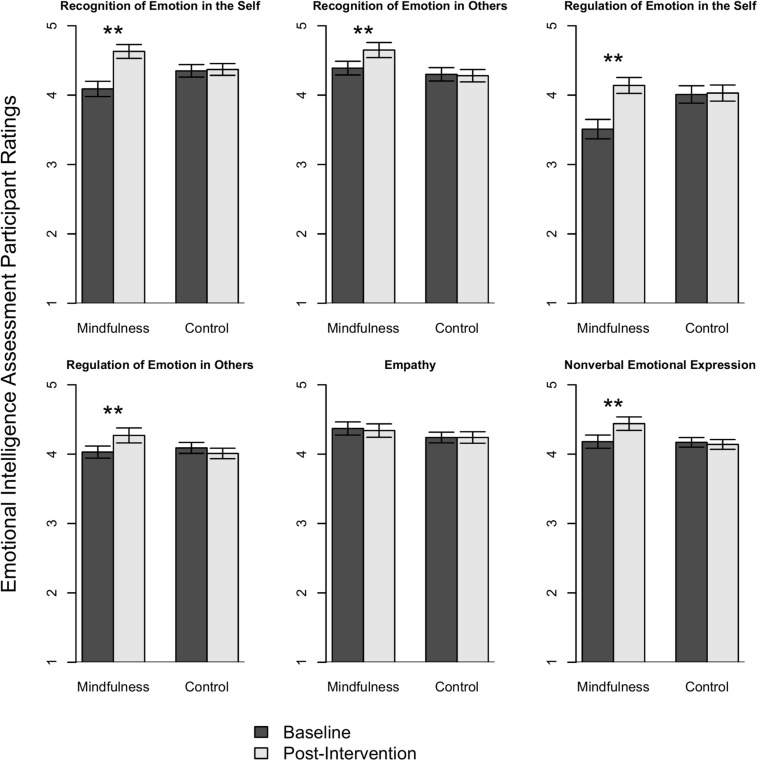
Emotional intelligence ratings. **p < 0.01.

We were interested in whether participants who participated in the mindfulness intervention differed from control condition participants on assessments of mindfulness, well-being, self-perceived EI, and workplace competencies following the intervention. Table 4 shows the means for each group on the mindfulness, well-being, and emotional intelligence assessments. To determine the effect of the intervention on the mindfulness, well-being, and EI outcome measures, a series of multivariate repeated measures analyses of covariance (MANCOVAs) were completed with pre- and post-intervention scores as dependent variables. Three analyses were performed: one with the trait mindfulness facets of the FFMQ-SF (observing, describing, non-reacting, non-judging, acting with awareness), one with the well-being-based outcome variables (perceived stress, resilience, positive mood, and negative mood), and one with the EI subscales of the MEIA-W (recognition of emotion in self, regulation of emotion in self, recognition of emotion in others, regulation of emotion in others, non-verbal emotional expression, and empathy). Table 4 shows the pre-and post-intervention means and standard deviations of the mindfulness and control conditions on the FFMQ-SF, well-being, and EI subscales and all pre- and post-intervention alpha coefficients for each scale/subscale. The MANCOVA with pre-and post-intervention mindfulness subscale variables set as dependent variables showed a significant within-subjects time × condition interaction (*F*_5_,_96_ = 5.28, *p* < 0.001, partial *n*^2^ = 0.22). The univariate tests revealed that the intervention group reported greater increases in all facets of the FFMQ-SF (all *p*’s < 0.05), with the exception of non-judgment (*p* > 0.05), partially supporting H1. The MANCOVA with pre-and post-intervention well-being-based outcome variables set as dependent variables also showed a significant time × condition interaction (*F*_4_,_97_ = 19.22, *p* < 0.001, partial *n*^2^ = 0.44). The intervention group reported greater reductions in stress (*F*_4_,_97_ = 75.74, *p* < 0.001, partial *n*^2^ = 0.43) and negative mood (*F*_4_, _97_ = 34.79, *p* < 0.001, partial *n*^2^ = 0.26), and greater increases in resilience (*F*_4_,_97_ = 37.35, *p* < 0.001, partial *n*^2^ = 0.27) and positive mood (*F*_4_,_97_ = 24.42, *p* < 0.001, partial *n*^2^ = 0.20) than the control group, supporting H2, H3, and H4 respectively, and these results can be seen in Figure 2. In support of H4a, reductions in self-reported negative mood were larger than improvements in self-reported positive mood. Finally, the MANCOVA with pre-and post-intervention EI outcome variables set as dependent variables showed a significant within subjects’ time x condition interaction (*F*_6_,_95_ = 5.64, *p* < 0.001, partial *n*^2^ = 0.26). Univariate tests revealed that the intervention group reported greater increases in all of the subscales than the control group (all *p*’s < 0.01) with the exception of empathy, which did not show any significant changes (*p* > 0.05), partially supporting H5. Full EI results are shown in Figure 3.

**TABLE 4:** Means, reliabilities, and change scores on mindfulness, perceived stress, resilience, affect, and emotional intelligence across conditions and timepoints.

	Mindfulness (*n* = 37)						Control (*n* = 65)						Change Scores				Univariate Test Results		
	BL			PI			BL			PI			Mindfulness		Control		Time × Condition Contrasts		
Variable	*M*	*SD*	α	*M*	*SD*	α	*M*	*SD*	α	*M*	*SD*	α	*M*	*SD*	*M*	*SD*	*df*	*F*	*Partial n^2^*
**FFMQ-SF**																			
Observing	13.65	3.46	0.81	15.65	2.71	0.76	14.22	3.56	0.80	14.19	3.63	0.82	0.50	0.80	−0.01	0.63	5, 96	12.63	0.11
Describing	17.32	4.07	0.87	19.49	3.57	0.85	17.49	4.06	0.85	17.69	3.72	0.84	0.43	0.56	0.04	0.58	5, 96	11.11	0.10
Non-judging	16.26	3.81	0.80	18.46	2.86	0.88	16.33	4.63	0.86	17.40	4.56	0.87	0.44	0.78	0.21	0.79	5, 96	1.98	0.02
Non-react	14.00	3.41	0.83	16.76	3.31	0.82	14.51	3.62	0.85	14.95	3.51	0.77	0.55	0.76	0.09	0.58	5, 96	11.99	0.11
ActAware	15.49	4.49	0.86	18.35	3.18	0.88	15.54	3.94	0.84	15.37	3.86	0.85	0.57	0.82	−0.03	0.66	5, 96	16.68	0.14
**Well-being**																			
PSS	26.35	7.60	0.89	17.57	5.40	0.90	23.22	8.24	0.88	24.34	7.96	0.89	−8.78	6.74	1.12	4.71	4, 97	75.74	0.43
BRS	3.29	0.77	0.86	3.78	0.69	0.89	3.63	0.71	0.88	3.41	0.75	0.86	0.48	0.68	−0.22	0.47	4, 97	37.35	0.27
PANAS_P	32.76	8.06	0.92	36.97	6.68	0.90	33.86	6.23	0.89	32.02	6.67	0.89	0.42	0.56	−0.19	0.61	4, 97	24.42	0.20
PANAS_N	22.32	6.09	0.87	16.84	4.76	0.88	21.43	7.75	0.90	21.75	7.15	0.88	−0.55	0.47	0.03	0.48	4, 97	34.79	0.26
**MEIAW**																			
RecSelf	4.09	0.67	0.74	4.63	0.61	0.87	4.35	0.72	0.77	4.37	0.69	0.81	0.54	0.55	0.03	0.48	6, 95	24.28	0.20
RecOther	4.39	0.60	0.76	4.65	0.66	0.86	4.30	0.78	0.84	4.28	0.72	0.86	0.26	0.43	−0.01	0.48	6, 95	18.82	0.16
RegSelf	3.51	0.85	0.89	4.14	0.70	0.89	4.01	1.01	0.89	4.03	0.94	0.88	0.63	0.83	0.03	0.57	6, 95	8.16	0.08
RegOther	4.03	0.53	0.69	4.27	0.66	0.81	4.09	0.64	0.83	4.01	0.61	0.79	0.23	0.43	−0.09	0.42	6, 95	13.70	0.12
Empathy	4.37	0.58	0.70	4.34	0.59	0.77	4.24	0.62	0.70	4.24	0.67	0.78	−0.03	0.42	0.00	0.39	6, 95	0.11	0.001
Non-verbal	4.18	0.58	0.59	4.44	0.59	0.77	4.17	0.56	0.65	4.14	0.57	0.63	0.26	0.45	−0.03	0.37	6, 95	12.28	0.11

*BL, Baseline; PI, Post-Intervention; FFMQ-SF, Five Facet Mindfulness Questionnaire – Short Form; PSS, Perceived Stress Scale; BRS, Brief Resilience Scale; PANAS_P, Positive Affect Negative Affect Schedule – Positive Affect Score; PANAS_N, Positive Affect Negative Affect Schedule – Negative Affect Score; MEIAW, Multidimensional Emotional Intelligence Assessment – Workplace; RecSelf, Recognition of Emotion in the Self; RecOthers, Recognition of Emotion in Others; RegSelf, Regulation of Emotion in the Self; RegOthers, Regulation of Emotion in Others; Non-verbal, Non-verbal Emotional Expression.*

#### Relationships Between Outcome Measures

A series of between group (intervention and waitlist control) bivariate Pearson *r* correlations were conducted between all mindfulness, well-being, and EI change scores (T2 – T1), shown in Table 5 along with alpha coefficients. There were several strong relationships in the intervention group. Changes in the FFMQ-SF facets acting with awareness and non-reactivity to inner experience demonstrated the strongest relationships with the well-being outcome variables, including a positive relationship between changes in acting with awareness and changes in resilience, *r*(37) = 0.60, *p* < 0.01, and between changes in non-reacting to inner experience with changes in resilience, *r*(37) = 0.66, *p* < 0.001. Changes in resilience were also associated with changes in positive mood, *r*(37) = 0.64, *p* < 0.01, and negative mood, *r*(37) = −0.68, *p* < 0.01. Changes in perceived stress were associated with changes in acting with awareness, *r*(37) = −0.56, *p* < 0.01, changes in non-reacting to inner experience, *r*(37) = −0.62, *p* < 0.001, and changes to regulation of emotions in the self, *r*(37) = −0.72, *p* < 0.01. Changes in positive mood were related to changes in non-reacting to inner experience, *r*(37) = 0.61, *p* < 0.001. Increases in self-perceived recognition of emotion in the self and regulation of emotion in the self correlated positively with changes in mindfulness facets, specifically changes in describing correlated most strongly with changes in self-perceived recognition of emotion in the self, *r*(37) = 0.67, *p* < 0.001, and changes in self-perceived regulation of emotion in the self, *r*(37) = 0.49, *p* < 0.01. Changes in non-reacting to inner experience correlated strongly and positively with changes in self-perceived recognizing emotion in the self, *r*(37) = 0.64, *p* < 0.001, changes in self-perceived regulation of emotions in the self, *r*(37) = 0.56, *p* < 0.001, and changes in self-perceived regulation of emotions in others, *r*(37) = 0.69, *p* < 0.001.

**TABLE 5:** Correlations between change scores and alpha coefficients.

	PSS	PANAS_P	PANAS_N	BRS	Describe	Non-react	Non-judge	Observe	ActAware	RecSelf	RegSelf	RecOther	RegOther	Empathy	Non-verbal
PSS	(0.90/0.89)	−0.758***	0.811***	−0.789***	−0.366*	−0.619***	−0.346*	−0.438**	−0.556***	−0.487**	−0.715**	−0.093	−0.371*	0.260	−0.322
PANAS_P	−0.261*	(0.91/0.89)	−0.615**	0.638**	0.294	0.606**	0.268	0.473*	0.437**	0.459**	0.461**	0.229	0.469**	−0.253	0.338*
PANAS_N	0.447***	−0.270*	(0.88/0.89)	−0.679**	−0.506**	−0.639**	−0.314	−0.407*	−0.565**	−0.501**	−0.662**	−0.203	−0.473**	0.080	−0.333*
BRS	−0.274*	*0.119*	−0.290*	(0.88/0.87)	0.305	0.660***	0.395*	0.302	0.599**	0.508**	0.735**	0.210	0.503**	−0.206	0.231
Describe	−*0.154*	0.247*	−*0.109*	*0.134*	(0.86/0.85)	0.449**	0.058	0.528**	0.465**	0.666**	0.488**	0.343*	0.512**	0.007	0.584**
Non-react	−*0.139*	*0.204*	−*0.215*	*0.088*	0.270*	(0.83/0.81)	0.071	0.417*	0.576***	0.642***	0.558***	0.237	0.688***	−0.323	0.396*
Non-judge	−0.327**	*0.210*	−*0.114*	−*0.033*	0.393**	*0.167*	(0.84/0.87)	0.031	0.173	0.192	0.331*	0.222	0.160	−0.144	0.163
Observe	−0.291*	*0.117*	−*0.029*	−0.267*	−*0.014*	*0.129*	*0.170*	(0.79/0.81)	0.561**	0.544**	0.459**	0.228	0.210	0.249	0.451**
ActAware	−*0.127*	0.330**	−0.349**	−*0.114*	0.262*	*0.210*	0.395**	*0.071*	(0.87/0.85)	0.488**	0.598**	0.315	0.589**	0.093	0.417*
RecSelf	*0.110*	−*0.068*	*0.104*	*0.140*	*0.126*	*0.057*	−*0.076*	−*0.238*	*0.107*	(0.81/0.79)	0.654**	0.286	0.641**	−0.073	0.589**
RegSelf	−*0.174*	*0.104*	−*0.076*	*0.217*	*0.174*	−*0.186*	*0.181*	*0.007*	*0.228*	0.491**	(0.89/0.89)	−0.057	0.400*	−0.145	0.363*
RecOther	*0.206*	−0.252*	*0.167*	*0.119*	−*0.243*	−*0.024*	−*0.184*	−*0.224*	−*0.093*	*0.121*	−*0.041*	(0.81/0.85)	0.481**	0.072	0.173
RegOther	*0.092*	*0.030*	*0.072*	*0.136*	−*0.077*	*0.016*	−*0.111*	−*0.036*	*0.048*	*0.225*	*0.132*	0.459**	(0.75/0.81)	−0.098	0.469**
Empathy	0.404**	*0.203*	*0.189*	−*0.064*	−*0.052*	*0.015*	−*0.120*	−*0.159*	−*0.092*	−*0.136*	−*0.196*	*0.061*	*0.023*	(0.74/0.74)	0.098
Non-verbal	*0.054*	*0.175*	−*0.043*	−*0.141*	*0.056*	−*0.027*	*0.009*	*0.169*	*0.171*	*0.192*	*0.099*	0.344**	*0.207*	*0.190*	(0.68/0.64)

Top of table is mindfulness condition (*N* = 37), bottom is control condition (italicized, *N* = 65). ^1^ PSS, Perceived Stress Scale; BRS, Brief Resilience Scale; PANAS_P, Positive Affect Negative Affect Schedule – Positive Affect Score; PANAS_N, Positive Affect Negative Affect Schedule – Negative Affect Score; RecSelf, Recognition of Emotion in the Self; RecOthers, Recognition of Emotion in Others; RegSelf, Regulation of Emotion in the Self; RegOthers, Regulation of Emotion in Others; Non-verbalExp, Non-verbal Emotional Expression. *p < 0.05; **p < 0.01; ***p < 0.001. Alpha coefficients presented along diagonal: averaged pre-post mindfulness condition presented first, averaged pre-post control condition presented second, full alphas across conditions and time shown in Table 4.

#### Changes in Workplace Competencies

Participants in the intervention condition completed a 27-item measure of workplace competencies, and selected colleagues to rate them on these same competencies pre- and post-intervention. We were interested in examining whether mindfulness training improved self-reported job performance. On the overall job performance index (consisting of the averaged scores across the 27 competencies), mean post-intervention scores (*M* = 5.31, *SD* = 0.61) were significantly higher than mean baseline scores (*M* = 4.84, *SD* = 0.59), *t*(36) = −5.50, *p* < 0.001, two-tailed. To explore the specific dimensions of job performance most impacted, we sorted the 27 competencies according to the Cohen’s *d* effect size of the improvement, shown in Table 6. The largest changes were found on Decisiveness, Making Tough Calls, Assuming Responsibility, Interpersonal Relationships, and Creativity, with Cohen’s *d* values ranging from moderate (*d* = 0.54) to strong (*d* = 0.76), and supporting H6 and H7. Given the number of indicators, we gave consideration to controlling family wise error rates in significance testing. Family wise error corrections, such as Bonferroni, are methods to control Type 1 errors (false positives) when performing multiple independent hypothesis tests. However, indicators in Table 6 are not independent, they are all related facets of a single over-arching construct: job performance. They are related tests of the hypothesis that mindfulness training improves job performance. Accordingly, Bonferroni corrections may be overly conservative leading to inflated Type 2 error rates (false negatives). Of the 20 competencies showing significant improvement with simple t-tests (*p* < 0.05), only 9 would still be significant after applying a Bonferroni correction (*p* < 0.002).

**TABLE 6:** Sample means and standard deviations for workplace competencies across groups and two timepoints: baseline (BL), postintervention (PI), sorted by Cohen’s *d* value.

Competency	*n*	BL	PI	Average change	Cohen’s *d*	*p*-value
**Decisiveness**	**37**	**4.59**	**5.46**	**0.86**	**0.76**	**0.0000**
**Making Tough Calls**	**36**	**4.06**	**4.72**	**0.67**	**0.66**	**0.0002**
**Assuming Responsibility**	**37**	**5.05**	**5.73**	**0.68**	**0.64**	**0.0002**
**Interpersonal Relationships**	**37**	**5.35**	**6.05**	**0.70**	**0.61**	**0.0003**
**Creativity**	**37**	**4.51**	**5.19**	**0.68**	**0.58**	**0.0005**
**Emotional Depth**	**37**	**4.51**	**5.16**	**0.65**	**0.58**	**0.0005**
**Intellectual Horsepower**	**37**	**4.43**	**5.08**	**0.65**	**0.56**	**0.0008**
**Vision**	**37**	**4.19**	**5.00**	**0.81**	**0.55**	**0.0009**
**Prioritizing**	**37**	**4.68**	**5.32**	**0.65**	**0.54**	**0.0012**
Persuasiveness	36	4.14	4.81	0.67	0.52	0.0019
Emotional Control	37	4.05	4.81	0.76	0.48	0.0032
Social Astuteness	37	4.35	4.92	0.57	0.47	0.0037
Mental Agility	37	4.59	5.24	0.65	0.46	0.0043
Work/Life Balance	37	4.49	5.14	0.65	0.42	0.0077
Listening	37	5.00	5.43	0.43	0.40	0.0107
Conflict Management	36	4.14	4.56	0.42	0.39	0.0132
Desire to Learn	37	5.68	6.00	0.32	0.38	0.0132
Objectivity	37	4.54	5.03	0.49	0.35	0.0212
Dependability	37	5.78	6.05	0.27	0.32	0.0288
Communication	36	5.03	5.36	0.33	0.30	0.0416
Integrity	37	6.32	6.51	0.19	0.26	0.0642
Open-Mindedness	37	5.27	5.57	0.30	0.24	0.0738
Independence	37	5.81	5.97	0.16	0.24	0.0801
Achievement and Motivation	37	5.62	5.81	0.19	0.20	0.1139
Thoroughness	37	5.22	5.43	0.22	0.19	0.1217
Productivity	37	5.41	5.59	0.19	0.18	0.1460
Negotiation	35	4.26	4.40	0.14	0.13	0.2320

*BL, Baseline; PI, Post-Intervention; *p* = one-tailed, bolded = significant after Bonferroni correction.*

Colleagues also rated intervention participants on the 27 competencies before and after the training program, and these results can be seen in Table 7. Although several competencies were rated more highly following the intervention, the overall job performance index was not significantly different following the intervention, *t*(36) = 0.91, *p* > 0.05, and H8 was not supported.

**TABLE 7:** Mean workplace competency ratings by colleagues, sorted by Cohen’s *d* value.

Competency	*n*	BL	PI	Average change	*SD*	Cohen’s *d*	*p*-value*
Thoroughness	17	5.90	6.41	0.51	0.53	0.96	0.001
Social Astuteness	17	5.22	5.79	0.58	0.81	0.71	0.005
Independence	17	6.01	6.52	0.51	0.73	0.70	0.006
Negotiation	14	4.95	5.67	0.71	1.13	0.63	0.017
Creativity	16	4.71	5.54	0.83	1.51	0.55	0.022
Intellectual Horsepower	17	5.75	5.98	0.23	0.43	0.53	0.023
Open-Mindedness	17	5.11	5.65	0.53	1.09	0.49	0.030
Emotional Control	17	4.85	5.45	0.60	1.24	0.48	0.032
Listening	17	5.52	5.90	0.38	0.84	0.46	0.039
Vision	16	5.18	5.78	0.60	1.35	0.45	0.047
Prioritizing	16	5.64	6.03	0.40	0.89	0.45	0.048
Decisiveness	17	5.49	5.81	0.33	0.77	0.42	0.049
Mental Agility	16	4.90	5.33	0.44	1.04	0.42	0.056
Achievement and Motivation	17	6.10	6.45	0.35	0.85	0.41	0.054
Communication	17	5.86	6.17	0.30	0.81	0.38	0.070
Objectivity	17	4.93	5.33	0.40	1.09	0.37	0.074
Dependability	17	6.26	6.52	0.25	0.77	0.33	0.095
Persuasiveness	17	5.08	5.56	0.48	1.51	0.32	0.106
Interpersonal Relations	17	5.61	5.98	0.37	1.18	0.31	0.108
Emotional Depth	17	5.21	5.55	0.34	1.08	0.31	0.108
Conflict Management	15	4.99	5.62	0.63	2.03	0.31	0.124
Work/Life Balance	17	5.59	5.90	0.31	1.22	0.26	0.153
Assuming Responsibility	16	5.84	5.99	0.15	0.84	0.17	0.248
Desire to Learn	17	5.97	6.09	0.12	0.98	0.13	0.307
Productivity	16	6.19	6.25	0.06	0.47	0.12	0.318
Making Tough Calls	16	5.46	5.43	−0.04	1.20	−0.03	0.453
Integrity	17	6.69	6.65	−0.04	0.56	−0.07	0.389

BL, Baseline; PI, Post-Intervention; *p = one-tailed.

## Discussion

The purpose of the present study was to investigate the effectiveness of an online 8-week mindfulness-based training program on a variety of mindfulness, well-being, self-perceived emotional intelligence, and workplace competencies in a sample of knowledge workers. Results showed that the intervention was successful, with participants reporting increases in mindfulness, resilience, positive mood, and self-perceived emotional intelligence, and decreases in stress and negative mood after participating in the program. Participants rated themselves higher on 27 workplace competencies following the intervention, but colleagues did not.

Trait mindfulness increased following the intervention, with the exception of the non-judging inner experience facet of the FFMQ-SF. Few prior organizational mindfulness interventions have employed the FFMQ or FFMQ-SF (Aikens et al., 2014; Querstret et al., 2018), but both of those studies reported that non-judgment increased following their interventions. It is possible that the current intervention did not emphasize non-judgment sufficiently to result in significantly increased ratings, or perhaps the relatively low levels of prescribed and reported mindfulness practice were insufficient at causing changes on this mindfulness facet. Recent research by Chin et al. (2019a), hypothesized that increases in non-judgment are what drives reductions in stress in mindfulness intervention participants. Since we reported a significant decrease in perceived stress in the current sample this explanation does not account for our findings. Given that several organizational mindfulness-based interventions do not report employing any mindfulness assessment (Davidson et al., 2003; Bazarko et al., 2013; Shonin et al., 2014; Chin et al., 2019b; Slutsky et al., 2019) further research with organizational interventions is required to explore how the different facets of mindfulness are affected by different interventions. In contrast to the findings of Querstret et al. (2018), we reported significant increases on the non-reacting to inner experience facet of the FFMQ-SF.

Participants in the current intervention reported significantly lower levels of stress following the mindfulness intervention, and significantly higher levels of resilience, in line with prior interventions (Klatt et al., 2009; Wolever et al., 2012; Bazarko et al., 2013; Aikens et al., 2014; Chin et al., 2019a). We also found increases in self-reported positive mood, and decreases in negative mood. As expected, people who completed the 8-week program reported larger reductions in negative mood relative to increases in positive mood, mirroring prior mindfulness research using the PANAS (Brown and Ryan, 2003; Davidson et al., 2003; Chambers et al., 2008). The larger impact on negative mood may be due to the fact that the PANAS assesses high arousal positive affect including excitement, and interest. A measure assessing low arousal positive mood states such as contentment may show different results, a possibility that future research should address. Although our intervention was 8-weeks long, it prescribed a lower amount of daily meditation practice than many of the prior interventions, suggesting that a low daily amount (around 10 min) of mindfulness practice can significantly reduce stress, enhance resilience, and improve mood, a finding that has also been reported in research with very brief mindfulness interventions (Zeidan et al., 2010).

The present research assessed participant self-perceptions of trait emotional intelligence, and significant increases in trait self-perceived EI were reported for all of the facets (recognition of emotion in the self, regulation of emotion in the self, recognition of emotion in others, regulation of emotion in others, and non-verbal emotional expression), with the exception of empathy. Because prior mindfulness research has not used this assessment, we are limited in our ability to draw connections between our results and prior research. The finding that participant self-perceptions of emotion in the self and regulation of emotion in the self were subject to the largest increases following the intervention dovetails with the correlations reported by Brown and Ryan (2003) between trait mindfulness and attention to and clarity of emotions, because participants felt they were more attuned to their own and other’s emotions following the intervention. The results show that in addition to enhancing the self-perceived recognition and regulation of emotions in the self, people who completed the mindfulness intervention were more likely to feel they were more likely to display how they were feeling through body language and facial expressions (i.e., non-verbal emotional expression), and more likely to feel they were paying attention to and attempting to regulate the emotions of others (i.e., regulation of emotion in others). Prior research has shown inconsistent effects of mindfulness training on empathy, with some studies reporting increases (Shapiro et al., 1998; Birnie et al., 2010), and others reporting null results (Beddoe and Murphy, 2004; Galantino et al., 2005). In the present research self-perceived trait empathy (the tendency to draw upon empathy at work), did not change following the intervention. It is possible that for mindfulness interventions to increase self-perceived empathy at work, more time or practice is required, or, that specific practices, such as compassion-based practices, are needed (Hildebrandt et al., 2017; Böckler et al., 2018). A compassion-based practice was included in the current mindfulness intervention but not until the final week of the intervention, which may have been too late, and this practice was not focused on but included as something optional for participants to explore, so it’s unlikely that many participants were exposed to the practice on a consistent basis.

This study also showed that mindfulness increased self-reported workplace competencies. Participants felt that they were better able to make decisions, work creatively, and relate to others, amongst other leadership qualities following the intervention. These results align with the predictions made in prior reviews about mindfulness in the workplace positively impacting decision-making and interpersonal relationships (e.g., Glomb et al., 2011; Good et al., 2016), and with prior research showing that mindfulness practices can enhance cognitive flexibility (Colzato et al., 2012; Ostafin and Kassman, 2012). However due to the use of self-report it must be emphasized that these results may be subject to response bias. Colleague ratings were collected to address concerns with potential self-report response biases; however, the results were limited, and the colleague raters were not blind – raters knew that their colleagues were participating in a mindfulness program, and this could also have led to a positive response bias in their ratings.

This study provides evidence that an online-based mindfulness training program can enhance mindfulness, well-being, self-perceived emotional intelligence, and self-reported workplace performance. Although promising, there are several limitations in this study. One limitation is that mindfulness and well-being measures were assessed using self-report measures only, and that participants may have guessed at the desired responses at the time of the second assessment. A further limitation is that the EI assessment used provides a measure of people’s self-perceptions of the tendency to use EI at work, and did not assess ability. Future research should investigate whether there is concordance between self-perceived and ability-based measures of EI in a workplace sample. In an attempt to increase the breadth and validity of the results, workplace competencies were assessed using self and other-reports of performance (via colleague raters of participants), however, as noted above the response rate of colleague raters was relatively low, and colleague raters were not blinded. Future research should seek a larger sample of both participants and colleague/other raters and efforts should continue to be made to utilize multiple modes of assessing the benefits of mindfulness at work. Bartlett et al. (2017) reported similar challenges with collecting a sufficient quantity of external ratings and thus it appears to be a challenging aspect of conducting mindfulness interventions in workplace settings. An experience-sampling methodology as employed by Chin et al. (2019b) may prove fruitful in future research. Another limitation is that follow-up results were not collected, so we do not know how long the benefits of the intervention may last. Follow-up ratings were not collected to reduce the demand on participants, who devoted a substantial time commitment to the study. A final limitation is that the attrition rate was relatively high, although comparable to other MBI’s relying on the completion of online surveys. For example, Hülsheger et al. (2013) reported a dropout rate of 49.8%. It should be noted that due to the timing of the study, post-intervention data were collected in mid-late December 2017, a very busy time of year at the organization, which likely reduced the likelihood of participants completing the approximately 20-min online post-intervention assessment. Kersemaekers et al. (2018) reported similar difficulties in attaining a sample of participants to complete their intervention in various organizations, and again, an experience-sampling methodology that allows participants to provide data throughout their time at work may circumvent some of these issues. In contrast to other recent work with lower attrition rates (Slutsky et al., 2019) we did not have an on-site mindfulness facilitator, we used an online only program, and data collection was done remotely. All of these factors may have reduced the motivation of participants to complete the program or to complete the post-assessments. Finding ways to enhance participation and a sense of connection via remote learning is a worthy consideration when designing future online mindfulness programs and interventions. Finally, those participants who stayed in the study and completed the intervention may have done so out of a belief that the mindfulness program would be helpful, and this may have affected study outcomes. Given that many of the participants had prior meditation experience this is a possibility. However, given that many people may have prior experience with mindfulness due to its popularity, and organizations do not limit participation in mindfulness training to meditation-naïve participants, we wanted to include all eligible participants in the present research regardless of prior meditation experience. However, this could have led to response biases in the data that inflated the improvements in outcome measures. That said, the effect sizes reported in this present study are not out of the range of those effect sizes reported in previous research.

In summary the present research provides support for the benefits of online mindfulness interventions in workplace settings, even for those participants who already have prior experience with mindfulness. Online mindfulness training can enhance mindfulness, well-being, self-perceptions of emotional intelligence, and workplace performance, and future research should continue to explore these benefits in diverse samples of people, in different kinds of organizations, and with diverse methods for collecting high quality data from both self and external sources.

## Data Availability Statement

The datasets generated for this study are available on request to the corresponding author.

## Ethics Statement

Informed consent was obtained from all individual participants in this study. All procedures performed in studies involving human participants were in accordance with the ethical standards of the University of Western Ontario Institutional Review Board and the American Psychological Association.

## Author Contributions

RN designed the 8-week online program and created the online program materials, assisted with designing the study, executed the study, completed the data analysis, and wrote the manuscript. JC assisted with designing the 8-week online program, designing the study, and supervised the data analysis and manuscript preparation. JM assisted with designing the study, and supervised execution of the study, data analysis, and manuscript preparation.

## Conflict of Interest

RN and JC are employed by SIGMA Assessment Systems but did not receive any financial renumeration for their involvement with this research study. The remaining author declares that the research was conducted in the absence of any commercial or financial relationships that could be construed as a potential conflict of interest.

## Footnotes

1 The online program can be viewed by contacting the first author for access.
